# Comparison of tissue tropism and host response to enteric and respiratory enteroviruses

**DOI:** 10.1371/journal.ppat.1010632

**Published:** 2022-07-05

**Authors:** Ines Cordeiro Filipe, Han Kang Tee, Julien Prados, Isabelle Piuz, Samuel Constant, Song Huang, Caroline Tapparel

**Affiliations:** 1 Department of Microbiology and Molecular Medicine, University of Geneva, Geneva, Switzerland; 2 Bioinformatics Support Platform, University of Geneva, Geneva, Switzerland; 3 Epithelix SAS Geneva, Geneva, Switzerland; University of California, Irvine, UNITED STATES

## Abstract

Enteroviruses (EVs) are among the most prevalent viruses worldwide. They are characterized by a high genetic and phenotypic diversity, being able to cause a plethora of symptoms. EV-D68, a respiratory EV, and EV-D94, an enteric EV, represent an interesting paradigm of EV tropism heterogeneity. They belong to the same species, but display distinct phenotypic characteristics and *in vivo* tropism. Here, we used these two viruses as well as relevant 3D respiratory, intestinal and neural tissue culture models, to highlight key distinctive features of enteric and respiratory EVs. We emphasize the critical role of temperature in restricting EV-D68 tissue tropism. Using transcriptomic analysis, we underscore fundamental differences between intestinal and respiratory tissues, both in the steady-state and in response to infection. Intestinal tissues present higher cell proliferation rate and are more immunotolerant than respiratory tissues. Importantly, we highlight the different strategies applied by EV-D94 and EV-D68 towards the host antiviral response of intestinal and respiratory tissues. EV-D68 strongly activates antiviral pathways while EV-D94, on the contrary, barely induces any host defense mechanisms. In summary, our study provides an insightful characterization of the differential pathogenesis of EV-D68 and EV-D94 and the interplay with their main target tissues.

## Author summary

Enteroviruses (EVs) are important human pathogens, associated with more than 20 clinical presentations. They replicate predominantly in the intestinal and/or respiratory mucosae. The respiratory EV-D68 can be considered an emerging virus because it caused an unprecedent outbreak in 2014, and contemporary isolates display increased virulence and novel neurotropic potential. The genetically related enteric EV-D94 is less common and its pathogenesis remains poorly defined, however, its infection has also been associated with neurological symptoms such as acute flaccid paralysis. To decipher the pathogenic mechanisms of these two viruses, we investigated their tropism and innate immunity induction in relevant human respiratory, intestinal and neural tissue culture models. Our results highlight the critical role of temperature in restricting EV-D68 tropism. Furthermore, using transcriptomic analysis, we identified key differences between respiratory and intestinal tissues, with the latter exhibiting higher cell proliferation and being more immunotolerant. More importantly, we could demonstrate the different strategies applied by EV-D94 and EV-D68 towards the host antiviral response, with EV-D68 strongly activating antiviral pathways and EV-D94, in contrast, inducing few host antiviral transcripts. This work identifies key differences in the pathogenesis of these representative respiratory and enteric EVs, which may contribute to the development of targeted antiviral therapies.

## Introduction

Enteroviruses (EVs) are among the most prevalent viruses worldwide. Their infections can cause a variety of symptoms, from a common cold to life-threatening complications. EVs are small non-enveloped viruses with an icosahedral capsid of around 30nm of diameter, that encloses a positive-sense single-stranded RNA genome [1]. The *Enterovirus* genus is divided into 15 species, based on the sequence identity of the VP1 capsid protein. Of these 15 species, 7 infect humans (*Enterovirus A* to *D* and *Rhinovirus A* to *C*) [1–3]. EV-D68 and EV-D94 both belong to the *Enterovirus D* species, a small but unique species, that recapitulates the diversity of the *Enterovirus* genus [4]. EV-D94 was first discovered in 2007, in samples from sewage in Egypt and from acute flaccid paralysis (AFP) cases in the Democratic Republic of the Congo [5]. Even though it has only been detected in African countries, seroprevalence studies have shown a high prevalence of anti-EV-D94 antibodies in the Finnish population [5]. Its worldwide circulation may therefore be underestimated. EV-D94 is known to be acid stable, with a broad cellular tropism, thus presenting the typical characteristics of an enteric EV [5]. On the other hand, EV-D68 is a respiratory virus and certainly the most popular genotype of the *Enterovirus D* species. It is acid labile and optimally grows at 33°C, as opposed to its enteric counterparts [6]. Due to its similarity with rhinoviruses (RVs), it was first classified as human RV-87 and only later re-classified as EV-D68 [7]. Since a widespread outbreak in USA in 2014, EV-D68 has been considered a public health threat due to its high morbidity in children, with cases of severe respiratory illnesses and neurologic conditions, described as acute flaccid myelitis [8–11]. Both EV-D94 and EV-D68 are known to use α-2,6 and α-2,3-linked sialic acid as receptors [12–14] while ICAM-5 has been proposed as an alternative receptor for EV-D68 [15].

EV-D68 and EV-D94 represent an interesting paradigm of EV heterogeneity. They are closely related, belong to the same species and share 74.1% nucleotide sequence identity across the genome. However, they display distinct phenotypic traits *in vitro* and *in vivo*. Hence, they provide a unique opportunity to better understand the differential tropism and pathogenesis of respiratory and enteric EVs. In this study, we characterized EV-D94 and EV-D68 infections using relevant human 3D tissues culture models. Viral pathogenesis is poorly apprehendable in cell lines or animal model. Immortalized or cancer cells are distantly related to the human tissues from which they derive and often induce emergence of cell-adapted viruses. On the other hand, animal models must be adjusted to be infected with human viruses due to the species barrier. The use of transgenic mice, mouse-adapted viruses and/or artificial infection routes can solve this problem but often leads to a pathogenesis different from that observed in humans [16–18]. Reconstituted human tissue culture models perfectly mimic the composition and architecture of human tissues. Compared to 2D cultures, they reproduce a higher number of *in vivo* features, such as the heterogeneous cell composition and access to nutrients and oxygen, as well as the generation of apical–basal polarity. At the single-cell level, the morphology, adhesion, differentiation, proliferation, survival and gene expression profiles are also closer to *in vivo* [16,19,20]. The recent increase in the complexity of these models by implementation of co-cultures, in particular with immune cells, makes them even more relevant. In this study, we used 3D cultures of respiratory, intestinal and neural origin to better characterize EV-D94 and EV-D68 infections and host response. We showed that a critical limitation of EV-D68 to invade neural and intestinal tissues under physiological conditions is its temperature sensitivity, as the virus replicates efficiently in these tissues at 33°C but not at 37°C. Regarding respiratory tropism, we highlighted that EV-D94 presents significant lower fitness than EV-D68 in respiratory tissues due to a restriction after the binding step of the replication cycle. Moreover, using transcriptomic analysis, we emphasized intrinsic differences between respiratory and intestinal tissues in absence or presence of infection by these two viruses, intestinal tissues presenting higher cell proliferation rate and being more immunotolerant than respiratory tissues. Finally, we demonstrated a tissue-independent pattern of immune response to each virus: EV-D94 barely induces innate immune response, while, on the contrary, EV-D68 strongly activates antiviral pathways. Altogether this work reveals fundamental differences between enteric and respiratory EV-Ds and their interplay with the host response in their main replication site.

## Results

### Differential replication and IFN-λ induction by EV-D68 Fermon and EV-D94 in respiratory, intestinal and neural tissue culture models

**Fig 1:**
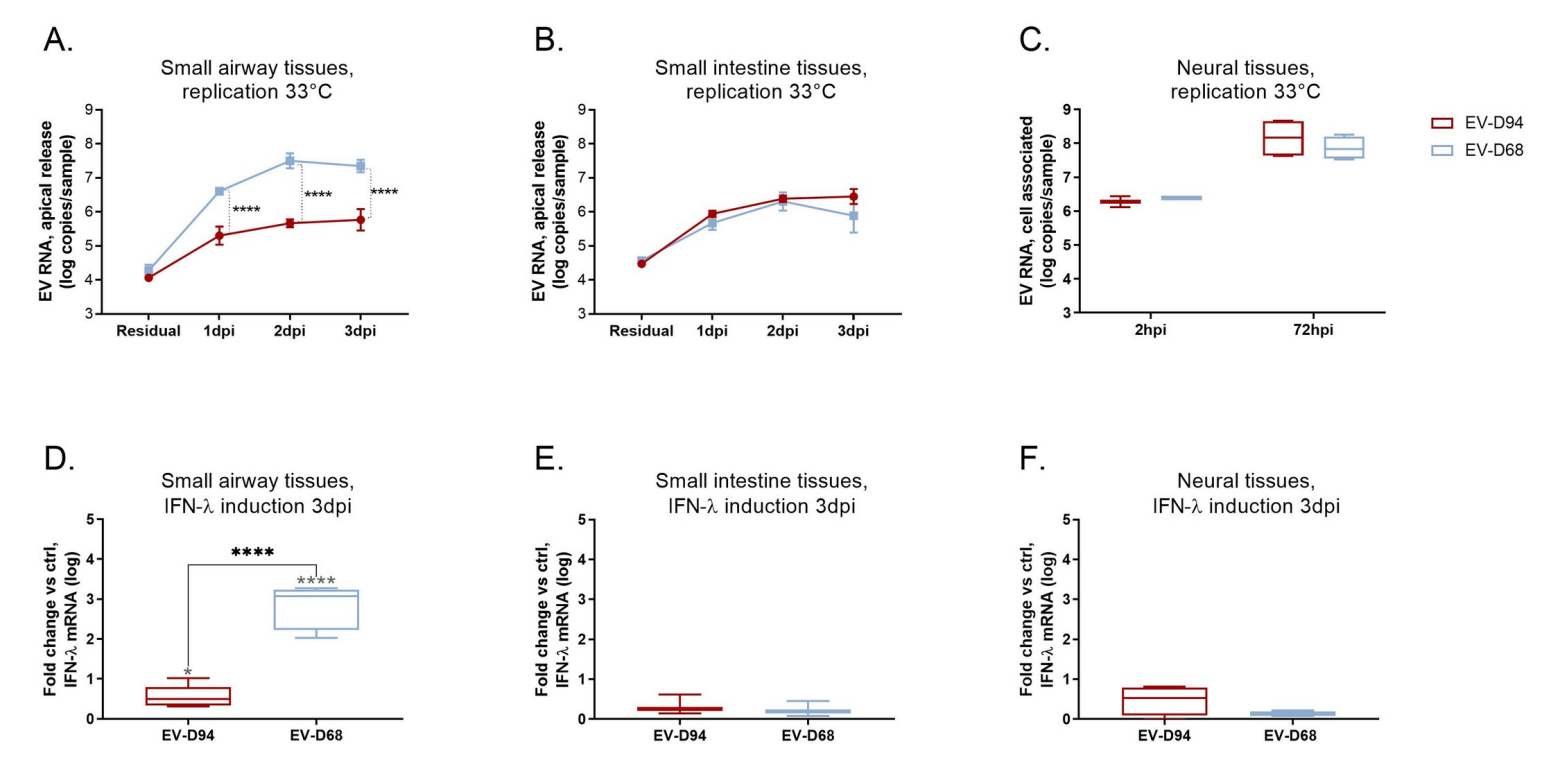
**EV-D68 and EV-D94 tissue tropism (A-C) and IFN-λ induction (D-F)**. Tissues were inoculated with equivalent multiplicity of infection (MOI) of EV-D94 and EV-D68 and replication was assessed by RT-qPCR. Respiratory (A) and intestinal (B) tissues were inoculated apically and washed 3 times at 3 hours post-infection (hpi). Residual virus was quantified after the washes. Apical samples were then collected at the indicated time points for viral RNA quantification. Neural tissues (C) were incubated with viral suspension and viral RNAs extracted from tissue lysate after inoculation (2hpi) and 3 days later were compared. IFN-λ mRNA levels (D-F) were measured in tissue lysates collected 3dpi and fold change relative to non-infected tissues was calculated with the ΔΔCt method. Significance between viruses is shown with black stars. In D, E and F, significance relative to mock-infected is shown with grey stars.

To investigate the determinants of EV tropism, we compared the replication of EV-D68 Fermon strain and EV-D94 in human 3D respiratory, intestinal and neural tissue culture models, which closely mimic their tissue of origin. In respiratory tissues derived from the small airways (SmallAir), EV-D68 produced significantly higher viral yields than EV-D94 (Fig 1A). This observation was confirmed in 2 other respiratory tissue culture models developed from human tracheal/bronchial epithelial cells (EpiAirway) and alveolar epithelial cells co-cultured with endothelial cells, fibroblasts and THP-1 macrophages (EpiAlveolar) (S1 Fig). On the other hand, in intestinal tissues we observed comparable fitness for EV-D68 and EV-D94, even though EV-D68 is not enterotropic *in vivo* (Fig 1B). Of note, we had previously shown that EV-D68 was not able to infect intestinal tissues, but in those experiments the infections were conducted at 37°C and not at 33°C as in the present work [21]. Similarly, in neural tissues, EV-D68 did not grow at 37°C [21], whereas in the current study the virus replicated at levels comparable to EV-D94 at 33°C (Fig 1C). These results suggest that the temperature sensitivity of EV-D68 is a major limitation to broader tropism *in vivo*.

Interferon lambda (IFN-λ) represents one of the first lines of host defense against viral infections at the mucosal surfaces and plays a key role in determining the outcome of the infection. Hence, we assessed IFN-λ induction by the two viruses in the respiratory, intestinal and neural tissues (Figs 1D–1F and S1). At 3 days post-infection (dpi), IFN-λ was significantly more induced by EV-D68 than by EV-D94 in respiratory tissues (Figs 1D and S1). This was particularly striking in the small airway model where IFN-λ levels were only slightly higher in EV-D94 infected tissues compared to non-infected controls (Figs 1D and S1B). Of note, the presence of THP-1 macrophages did not increase further the IFN response (S1 Fig). Surprisingly, this cytokine was not significantly induced in neural and intestinal tissues despite efficient replication of both viruses in these models (Fig 1E and 1F).

### EV-D94 is significantly less fit than EV-D68 in respiratory tissues due to a restriction after the binding step of viral life cycle

**Fig 2:**
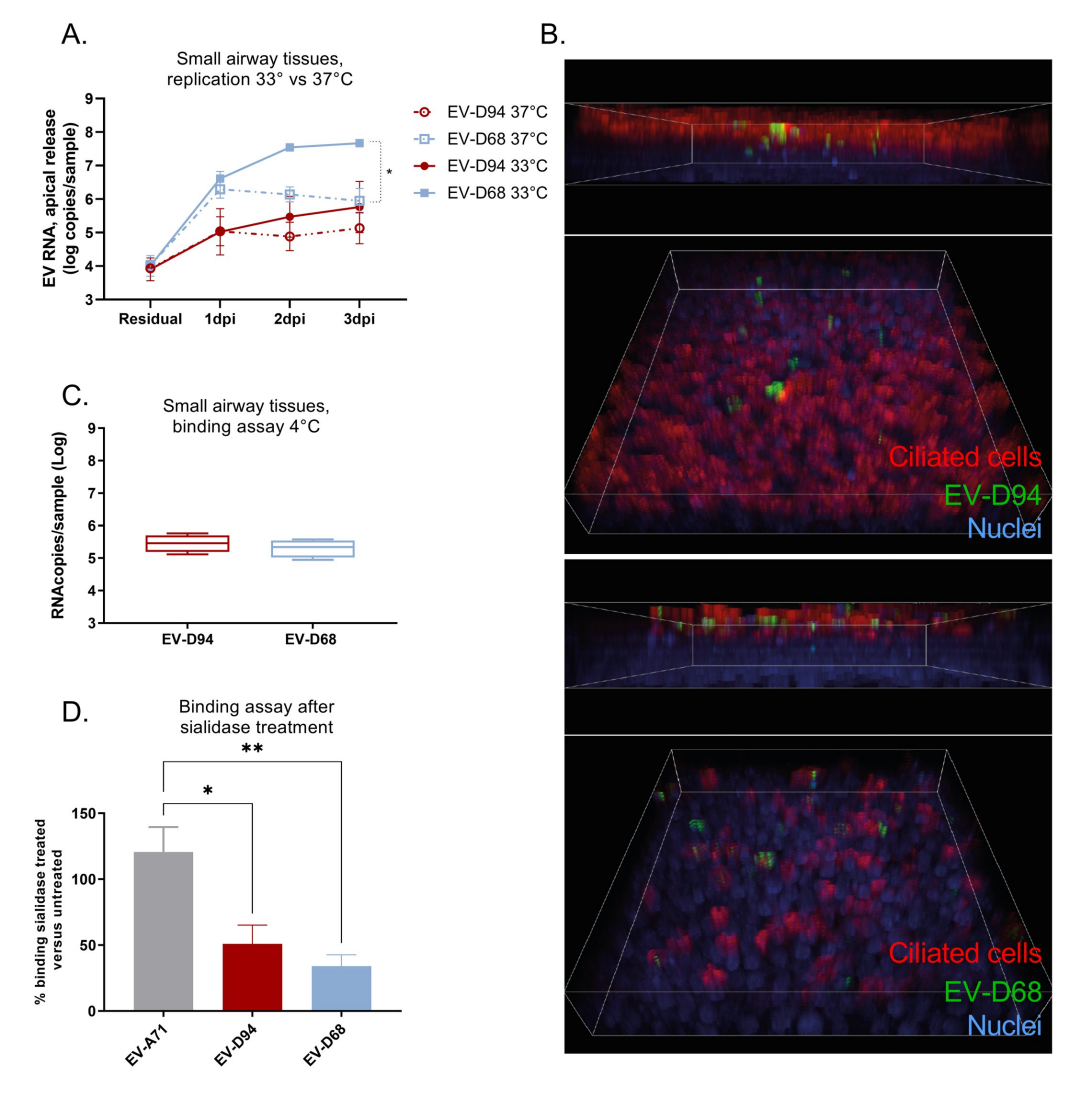
Differential replication of EV-D94 and EV-D68 in respiratory tissues. A) Viral replication at 33°C and 37°C. Viral replication was quantified as in Fig 1A and 1B) 3D projections of confocal images of infected respiratory tissues at 1dpi. Nuclei were stained with DAPI (blue), viral RNA with J2 antibody (green) and ciliated cells with anti-beta IV tubulin antibody (red). Images were acquired in the same area at different z-stacks and 3D projections (with apical tissue side on top) were generated with Imaris. The corresponding 2D images are available in S2 and S3 Figs with co-staining of virus and ciliated or basal cells respectively. C) Binding assay. Tissues were infected 1h 4°C, washed extensively and viral loads were quantified in tissue lysates by RT-qPCR. D) Binding assay in tissues pretreated or not with sialidase. Tissues were washed 3 times with PBS and incubated for 1h at 37°C with sialidase or with control buffer. After extensive washing, binding assay was run as in C. Treated and untreated tissues were processed in parallel for sialic acid staining (S4 Fig).

Amongst the three tissue culture models, the respiratory model is the only one presenting different susceptibility to EV-D68 and EV-D94 at 33°C. As EV-D94 is expected to grow optimally at 37°C, we first checked the impact of temperature on the growth of the two viruses in respiratory tissues (Fig 2A). As expected, EV-D68 replication was dramatically impaired at 37°C while EV-D94 infection was comparable at 37°C and 33°C. Of note, EV-D94 stock was produced at 33°C in HeLa cells, reaching titers similar to EV-D68 in this cell line. This confirms that in contrast to EV-D68, EV-D94 can rapidly adapt to 33°C as previously shown [21] and that temperature is not responsible for the different fitness of EV-D68 and EV-D94 in the respiratory model.

Subsequently, we hypothesized that EV-D68 and EV-D94 could infect different cell types in respiratory tissues, resulting in different infection kinetics. EV-D68 is known to infect ciliated cells and, after tissue injury, undifferentiated cells [22] while the respiratory cell tropism of EV-D94 has not been characterized yet. Thus, we performed dual labelling of infected tissues, at 1dpi, with antibodies targeting the virus and ciliated (Figs 2B and S2) or basal cells (S3 Fig). For EV-D94, as for EV-D68, the infection was restricted to the apical side of the tissue, in the ciliated cell layer (Figs 2B and S2). We also observed a clear difference in the cytopathic effect caused by each virus, with evident ciliated cell loss 1dpi with EV-D68 and no obvious change after infection with EV-D94 (Fig 2B). Therefore, the different viral kinetics exhibited by EV-D68 and EV-D94 are not the consequence of a different targeted cell population.

To determine whether the lower fitness of EV-D94 in respiratory tissues could be due to impaired binding, we compared amounts of cell-associated viruses 1h post-attachment at 4°C, but observed no significant differences between the two viruses (Fig 2C). In addition, we observed a significant reduction in binding for both EV-D94 and EV-D68 in tissues pretreated with sialidase, while a sialic-acid-independent EV-A71 variant was unaffected by the treatment (Fig 2D). This confirms that both variants use sialic acid to attach to the cells. Sialidase cleavage was confirmed by immunostaining (S4 Fig).

We then investigated if EV-D94 entry could be somehow delayed in respiratory tissues. We thus prepared EV-D94 and EV-D68 viral stocks in presence of neutral red, a dye known to crosslink viral RNA genome upon illumination, inhibiting viral uncoating. If viruses have not undergone uncoating before the light reaction, no new virus will be synthesized, whereas uncoated viruses will initiate a replication cycle. We inoculated small airway tissues with the neutral red labelled viruses and exposed them to light directly after inoculation, 1h, 2h or 3h post-inoculation and then quantified viruses at 48hpi by real-time quantitative RT-PCR (RT-qPCR). Both viruses were able to uncoat within the 1^st^ hour post-inoculation as they were fully protected upon exposure at this time point (S5 Fig). This suggests that EV-D94 has no delayed entry but rather that the block occurs at a post-entry stage of the life cycle.

**Fig 3:**
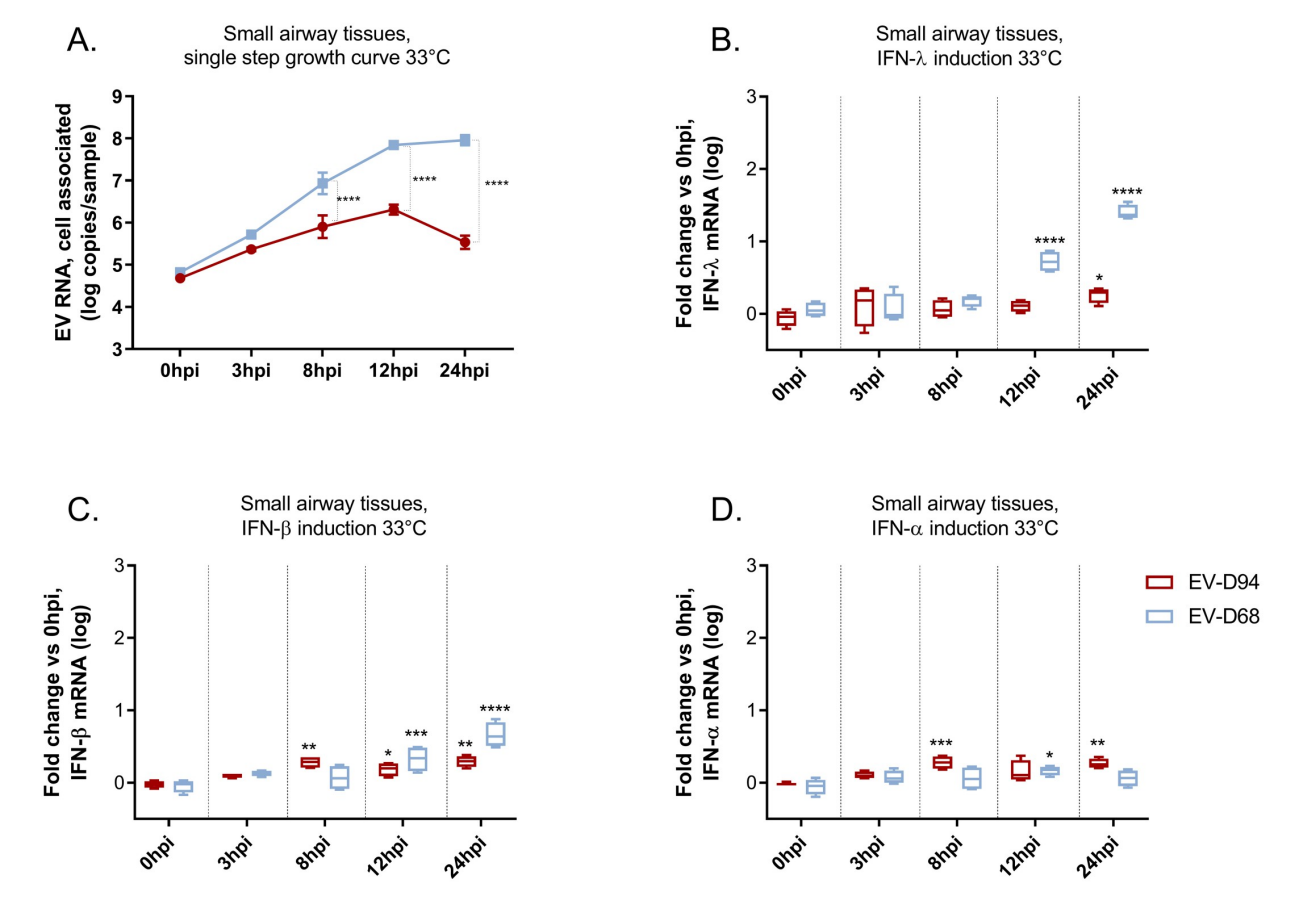
Single step growth curve assay of EV-D68 and EV-D94 grown at 33°C. Viral loads were quantified in tissue lysates by RT-qPCR at the indicated time points (A). IFN-λ (B), IFN-β (C) and IFN-α (D) mRNAs were quantified from the same tissue lysates as in A) and fold changes were calculated relative to the average induction observed for the two viruses at the time of infection (0hpi) with the ΔΔCT method. In A) Statistical analysis was performed comparing at each time point the replication between the two viruses while for B to D, significance was calculated at each time point, relative to IFN induction by the same virus at 0hpi.

Finally, we performed a single-step growth curve assay, quantifying viral loads at 3hpi, 8hpi, 12hpi and 24hpi (Fig 3A). We observed a significant difference between the two viruses at 8hpi, after replication onset. In parallel to viral loads, we quantified IFNs mRNA levels at the same time points (Fig 3B–3D). We did not observe significant IFN-λ induction by EV-D94 before 24hpi, indicating that IFN-λ expression is not the cause of the reduced replication observed before this time point. Interestingly, at 8hpi, EV-D94 induced a low but consistent 2-fold type I IFN (IFN-α and -β) response, while EV-D68 did not induce neither type I nor type III IFN before 12hpi. Accordingly, early induction of low levels of type I IFNs by EV-D94 may limit viral replication during the initial stage of the growth cycle. Furthermore, we detected a significant decrease in cell-associated EV-D94 at 24hpi concomitant with an increase of both type I and type III IFN response. EV-D68 on the other hand induced higher levels of IFN-β and IFN-λ starting at 12hpi, but appeared to be little affected because it continued to replicate at high levels thereafter.

### Respiratory and intestinal tissues present basic differences in absence of infection

Respiratory and intestinal tissues are the main replication sites of respiratory and enteric EVs, respectively. To further understand the different strategies applied by these 2 groups of viruses and their interplay with the host, we performed transcriptomic analysis of these two tissue models in absence or presence of infection by EV-D68 or EV-D94.

**Fig 4:**
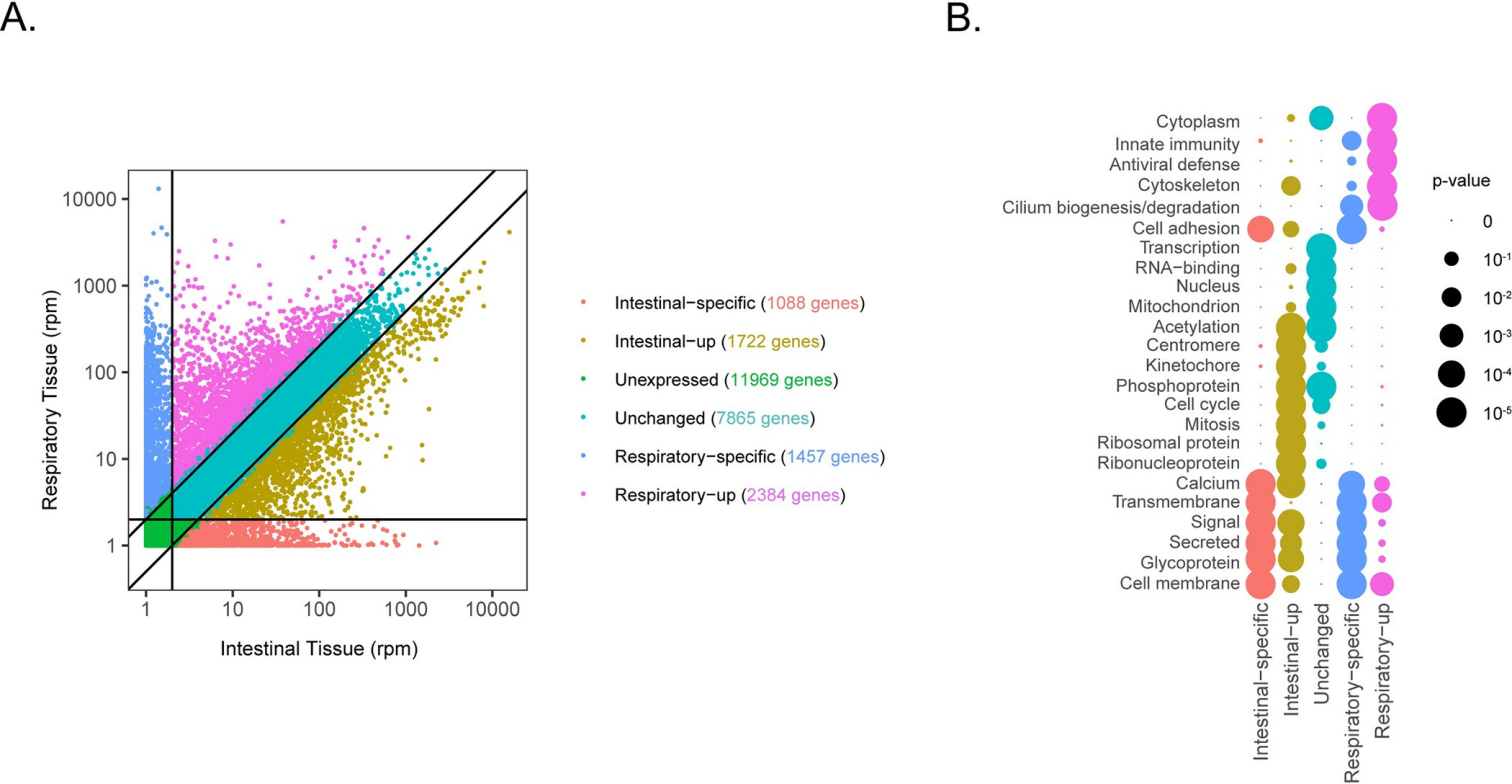
Comparison of the transcriptomic profile of non-infected respiratory and intestinal tissues. A) Gene expression in RNA reads per million (rpm) of mock-infected respiratory and intestinal tissues. A threshold of 1rpm determines expressed genes (horizontal/vertical lines). 2-fold changes determine “tissue-up” genes. Expressed genes were divided in 5 groups: orange (intestinal specific transcripts), brown (transcripts highly expressed in intestinal tissues), turquoise (transcripts equally expressed in both tissues), pink (transcripts highly expressed in respiratory tissues), blue (respiratory specific transcripts). Unexpressed genes in both tissues (‹1 rpm) are represented in green. B) Functions associated to each group of genes in A). Functions were defined by the top enriched uniport keywords associated to each group of genes. Circle sizes represent *p-value*. The list of genes for all categories is provided in the GEO database with accession number GSE184488.

We started by looking at the transcriptomic profile of each model in mock-infected controls (Fig 4). Both tissues expressed to similar extent genes related to transcription and to mitochondrial and nuclear functions, while transcripts related to membrane composition, glycoproteins, cell adhesion and calcium signaling showed tissue-specific signatures. Of note, genes involved in cell cycle progression and mitosis showed higher expression in intestinal tissues, reflecting the high proliferation rate of the intestinal epithelium [23,24]. Concerning respiratory tissues, transcripts related to cytoskeleton and ciliogenesis were enriched, which is in line with the large number of ciliated cells lining the respiratory mucosae. Interestingly, genes related to innate immunity and antiviral defense also showed higher basal expression in respiratory tissues, suggesting that these tissues are less immunotolerant than intestinal tissues.

### Respiratory and intestinal tissue response to EV-D68 and EV-D94 infection

**Fig 5:**
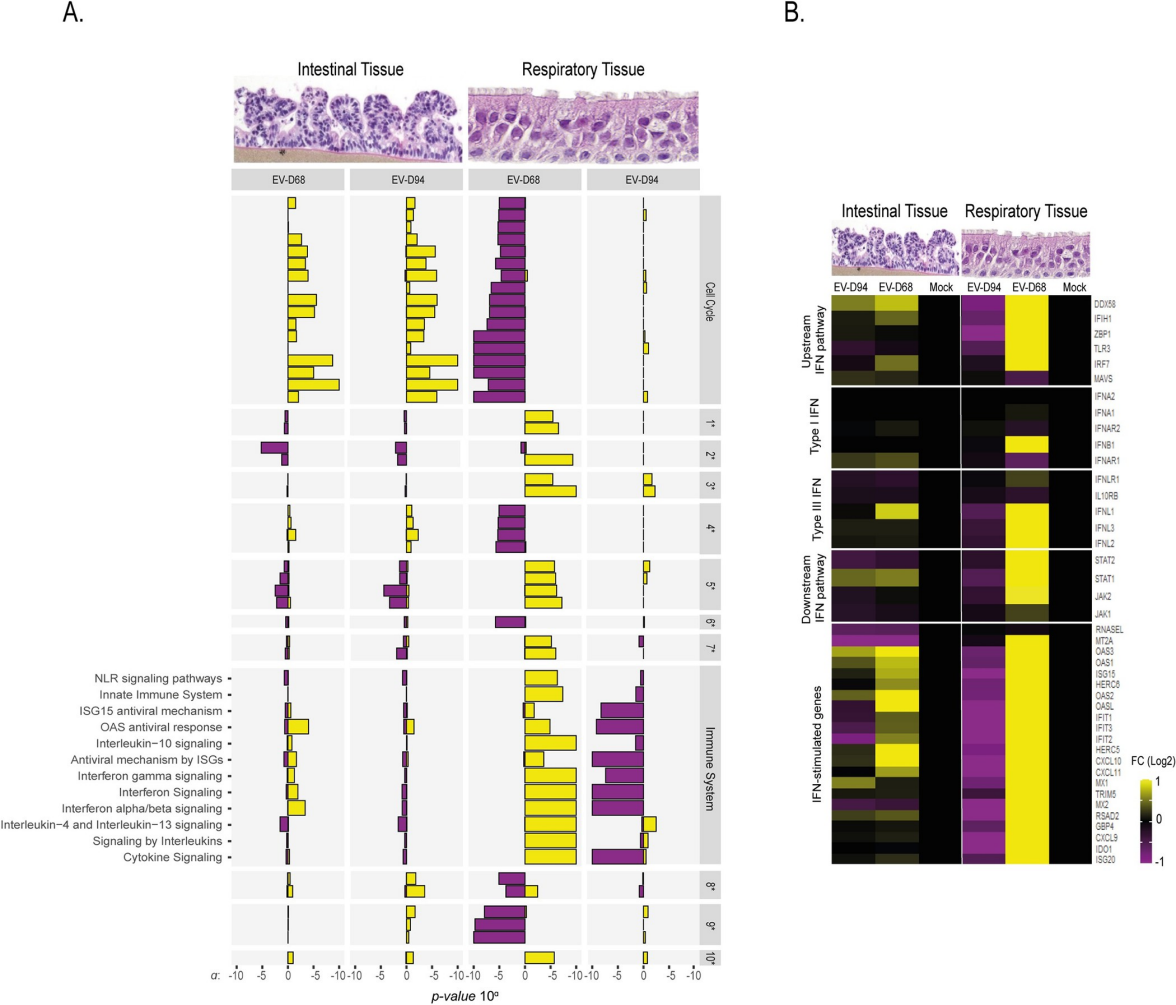
Significantly enriched pathways after infection by EV-D94 or EV-D68 in respiratory and intestinal tissues. **A)**. Genes with average log2 fold change increase/decrease of 0.5 versus the mock were considered for pathway analysis. Pathway with p-value < 0.00001 were classified as enriched. Yellow bars represent upregulated pathways while purple bars represent downregulated ones. Bar size illustrates p-value. 1* Cell-Cell communication; 2* Cellular responses to external stimuli; 3* Developmental biology; 4* DNA repair; 5* Extracellular matrix organization; 6* Gene expression (Transcription); 7* Hemostasis; 8* Metabolism; 9* Organelle biogenesis and maintenance; 10* Signal transduction. Functional annotations were obtained from reactome.org. **B) Heatmap of selected immune genes.** The FC values for each transcript are represented in S7 Fig. Images depicting intestinal and respiratory tissue sections were obtained from Mattek and from [25]. Another representation of these data is available in S6 Fig and at [http://genebrowser.unige.ch/enterovirus/].

Next, we analyzed the transcriptomic profile of tissues infected with the enteric (Figs 5 and S6A and S6B) or respiratory (Figs 5 and S6C and S6D) EV at 2dpi. EV-D94 infection altered numerous transcripts in intestinal tissues (Figs 5 and S6B), compared to respiratory tissues (Figs 5 and S6A), with the majority being up-regulated, independent of their function. Similarly, mostly upregulated transcripts were observed in EV-D68-infected intestinal tissues (Figs 5 and S6D). Of note, EV-D94 infection induced a stronger transcriptional perturbation, despite slightly lower amount of cell-associated virus (7.23 EV94 RNA copies (in log) versus 7.97 EV68 RNA copies (in log), S8 Fig).

In respiratory tissues, where EV-D68 replicated extensively, the virus induced higher and more diverse transcriptomic changes than in intestinal tissues (Figs 5 and S6C and S6D). Transcripts related to tissue metabolism, cell cycle progression, organelle biogenesis and maintenance, DNA replication and transcription were all downregulated, whereas genes and pathways related to immune response were upregulated. Regarding EV-D94, it induced little changes in respiratory tissues (Figs 5 and S6A) but the trend was towards downregulation of several innate immunity genes, resulting in specific and significant downregulation of immune pathways compared to mock-infected tissues (Fig 5B). In particular, IFNs, major RNA sensors such as RIG-I (DDX58), MDA5 (IFIH1) and TLR3 were downregulated or expressed to similar levels as mock-infected tissues, as were several ISGs such as ISG15, IFIT1, IFIT2, IFIT3, MX1, STAT1 (Figs 5B and S7 and S8). By contrast, EV-D68, in line with its high replication level, strongly upregulated the same sensors and ISGs (Figs 5B and S7 and S8). These transcriptome data were confirmed by RT-qPCR and western blot (S8A Fig).

To assess the impact of viral replication levels on innate immunity induction, we lowered the inoculum dose of EV-D68 by 100-fold, 1000-fold and 5000-fold. As shown in S9A and S9B Fig, infection with 1E4 RNA copies (3,3E2 TCID50) of EV-D68 did not decrease significantly the replication kinetics nor the intracellular RNA measured at 2dpi, confirming the high fitness of this virus compared to EV-D94 in respiratory tissues. When decreasing the inoculum to 5E3 RNA copies (165 TCID50), we observed a significant reduction in viral growth, below that observed upon infection with 1E7 RNA copies (2,46E4 TCID50) of EV-D94. We then assessed the tissue innate immune response and observed a correlation between viral loads at 2dpi and innate immunity induction (S9B and S9C Fig). However, and in line with our transcriptome results, EV-D94 induced almost no IFN nor ISG compared to mock-infected respiratory tissues, and induction levels were comparable to those observed upon infection with 1E3 RNA copies of EV-D68, although the latter showed lower viral yield in tissues at this time point (S9A and S9B Fig).

Finally, in intestinal tissues, the effects were less marked and no immune pathways were significantly enriched. However, the trend was conserved for each virus, with low-induction to down-regulation of immune pathway-related genes by EV-D94 and up-regulation by EV-D68 (Figs 5 and S7 and S8B), despite comparable intracellular RNA loads in intestinal tissues at this time point (7.23 log EV94 RNA copies versus 7.97 log EV68 RNA copies, S8B Fig). The lower impact of infection on immune pathways in intestinal tissues is probably a consequence of the reduced level of inflammation in these tissues in steady state conditions as mentioned above.

## Discussion

EV-D68 and EV-D94 are two distinct genotypes of the EV-D species. They are closely related at the genetic level, but present different *in vivo* tropism and pathogenic features. Here we used these two viruses as representatives of respiratory and enteric EVs. We first showed that both viruses grow to similar extent in intestinal and neural tissues. This is surprising as we used the EV-D68 Fermon strain, isolated in the 60s and described to present a restricted respiratory tropism [26]. In addition, this contradicts our previous study where EV-D68 only grew in respiratory tissues [21]. Interestingly the main difference between the two studies is the infection temperature, reduced from 37°C to 33°C in the current work. Rosenfeld and colleagues also showed that historic EV-D68 strains, including Fermon, can successfully infect neuroblastoma cells, IPS derived cortical neurons and organotypic mice brain tissues [27]. Similarly, their infections were also run at 33°C [27]. Therefore, tolerance to higher body temperatures certainly represents a critical prerequisite for CNS invasion, which is supported by the lower temperature sensitivity of recent EV-D68 neurotropic strains [28]. Of note, contemporary EV-D68 strains also exhibit greater acid tolerance [28]. Whether this would allow the virus to survive the acidic environment of the gastrointestinal tract *in vivo* remains to be determined. EV-D68 genomes have been detected in stool samples, but infectious viruses have not been recovered from the digestive tract yet [29]. The ability of old EV-D68 strains to grow in intestinal tissues at 33°C seems to depend on the tissue culture model. Freeman et al recently showed that historical EV-D68 strains do not replicate in stem cell-derived enteroids [28]. However, in this model, the apical tissue side, where the infection occurs, is enclosed within the organoid, making it hardly accessible for the virus.

Concerning EV-D94, we observed efficient replication in intestinal and neural tissues both at 33°C and 37°C [21]. Little is known about the pathogenesis of EV-D94, but because the virus was first isolated from a child with AFP [30], it is presumed to have neurotropic potential, like other enteric EVs. Enteric EVs are known to spread preferentially via fecal-oral route and replicate in the gastrointestinal tract, but they can be isolated from the respiratory tract and transmitted via respiratory droplets [31,32]. We therefore compared the respiratory tropism of EV-D94 and EV-D68 and highlighted that EV-D68 was consistently fitter than EV-D94 in respiratory tissues. This observation was confirmed in 3 different models of respiratory tissues, mimicking the upper respiratory tract, the small airways and the alveoli. Contrarily to EV-D68, the growth temperature did not affect EV-D94 tissue tropism. This is in line with our previous study, where we demonstrated that EV-D94 viral stock produced at 33°C was able to rapidly adapt to 37°C, which was not the case for EV-D68 [21].

We next attempted to define at which stage of the growth cycle EV-D94 undergoes fitness loss compared to EV-D68 in respiratory tissues. We observed no significant difference at the binding or entry step of the life cycle. This correlates with the fact that both viruses use sialic acid as receptor, a fact confirmed by the diminished binding observed in tissue predigested with sialidase. In addition, we showed that EV-D94 colocalizes with ciliated cell markers, as does EV-D68. Significant differences in viral loads were observed only from 8hpi, after RNA replication onset, and a further decrease was observed for EV-D94 at 24hpi. We investigated a possible implication of the host antiviral defense in the reduced replication of EV-D94 by measuring type I and type III IFN production at early times post-infections. No IFN-λ was induced by EV-D94 before 24h, a time point where viral replication decreased. However, we observed a consistent 2-fold induction of IFN-α and -β at 8hpi, in line with the timepoint where the fitness difference started to appear. Interestingly type I IFNs were not induced by EV-D68 at this time point despite higher replication. The antiviral activity of type I IFN may thus partly account for the restricted replication of EV-D94 at the very early stages of the replicative cycle while inhibition by type III IFN may occur later, starting at 24hpi. Interestingly, IFN induction by EV-D68 was much higher from 12hpi, but it did not impact viral replication which progressed rapidly despite this IFN response. So far, our data indicate that EV-D94 can bind and infect respiratory ciliated cells, similar to EV-D68, while it presents a disadvantage at a post-entry step of the replication cycle. Although type I interferon may interfere with early EV-D94 replication, additional investigations are needed to dissect the precise mechanism behind this tissue-specific restriction.

To further understand the interaction between EVs and their main target tissues, we performed a transcriptomic analysis of respiratory and intestinal tissues infected or not with EV-D94 and EV-D68. In absence of infection, we highlighted fundamental differences between the intestinal and respiratory models. Pathways enriched in intestinal tissues highlighted a high proliferation rate, while transcripts enriched in respiratory epithelia reflected the abundance of ciliated cells lining the respiratory mucosae. Most interestingly, genes related to innate immunity and antiviral defense also showed higher basal expression in uninfected respiratory tissues compared to intestinal tissues, indicating a higher basal level of inflammation.

After infection, transcriptomic alterations in intestinal tissues followed the same trend, regardless of virus. Many genes were upregulated by both viruses, independent of their function. In contrast, the response to EV-D94 and EV-D68 infection was very different in respiratory tissues. EV-D68 replicated strongly, inducing a shutoff of host metabolic pathways and a strong antiviral response. IFN-λ induction was already detected at 12hpi and increased by 3 logs relative to mock-infected tissues at 3dpi. Interestingly, EV-D68 replication stayed high despite this robust IFN-λ and ISGs expression. EV-D94 on the other hand induced low antiviral response up to 3dpi in the small airway respiratory model. In the transcriptomic analysis of these tissues infected for 2 days, we even observed that expression of major viral sensors, such as RIG-I, MDA5 and TLR3 as well as numerous ISGs remained at a comparable level or were even downregulated compared to mock-infected tissues. An observation confirmed by RT-qPCR and western blot. Of note, reducing the EV-D68 inoculum up to 1E4 RNA copies did not significantly change viral replication and innate immunity induction at 2 dpi while infection with 5E3 RNA copies of EV-D68 led to comparable viral loads and innate immunity induction by the two viruses. In intestinal tissues, immune pathways were not significantly enriched, which is in line with our observations that these tissues are intrinsically more immunotolerant and also with recently published results on EV-D68 [28]. In human intestinal epithelial cells, pattern recognition receptors (PRRs) are localized at the basolateral tissue side, correlating with tolerance of commensals present at the apical tissue side. Accordingly, viral infections originating from the basolateral side elicit stronger IFN response than infection from the luminal tissue side [33]. As we inoculated both EV-D94 and EV-D68 apically to mimic the natural infection route, this may explain their poor detection by immune sensors in intestinal tissues at early time post-infection. Nevertheless, the trend was maintained for each virus with induction of immune genes by EV-D68 and down regulation by EV-D94, despite comparable viral loads in this model. Interestingly, despite impairing host innate immunity, EV-D94 remained less or as fit as EV-D68 in respiratory and enteric tissues. This suggests that EV-D94 is globally less adapted than EV-D68 and that its replication would be even lower in the absence of this ability to interfere with the host antiviral response. As we did not observe similar shutdown of immune pathways upon EV-D68 infection despite high replication, the respiratory EV is probably less sensitive to the antiviral response or applies a different strategy to counter it, a strategy not observable at the transcriptome level, such as cleavage of downstream effectors. Of note, ruxolitinib (a JAK1/2 inhibitor) treatment of primary human bronchial epithelial cells increased EV-D68 replication, confirming that IFN pathways can indeed restrict EV-D68 replication [28]. Altogether, our data highlight that the respiratory and the enteric EV-D adopt different pathogenic strategies: EV-D68 triggers a strong IFN and ISGs response which does not prevent the virus from reaching high titers while EV-D94 prevents the host from establishing such strong antiviral response. Of note, other enteric EVs have been shown to trigger type III IFNs in enteroids, indicating strain-dependent strategies to combat host defenses [34,35].

In conclusion, our study provides an insightful characterization of EV-D68 and EV-D94, as surrogates for respiratory and intestinal EVs, as well as their main target tissues. We highlighted the key role of temperature and antiviral response in modulating viral replication. We showed that the two viruses have developed different strategies to successfully infect respiratory or intestinal tissues. At the same time, these tissues also have their own characteristics, further emphasizing the ability of these viruses to adapt to different environments and to disseminate into multiple tissues. In particular, EV-D68 has historically been classified as a respiratory virus, but has recently re-emerged as a neurotropic virus and possibly, in the future, as an enterotropic virus. Although EVs include several important human pathogens, there is currently no vaccine or antiviral available. Further studies are needed to fully understand the diversity of EV pathogenesis in order to develop effective antiviral strategies.

## Materials and methods

### Ethics statement

Respiratory tissues derived from the small airways (SmallAir) were ordered from Epithelix [https://www.epithelix.com/products]. Respiratory tissues derived from the upper respiratory track (EpiAirway) and from the alveoli (EpiAlveolar) as well as tissues derived from the small intestine (EpiIntestinal) were ordered from Mattek [https://www.mattek.com/products]. Neural tissues derived human pluripotent stem cells were ordered from Neurix [https://www.neurix.ch/]. The tissues were developed from anonymized samples and after ethical approval. The study was conducted according to the Declaration of Helsinki on biomedical research (Hong Kong amendment, 1989), and the research protocol was approved by our local ethics committee (CCER, study number N°2016–01122).

### Cell

HeLa Ohio cells (kindly provided by F. H. Hayden, University of Virginia, USA) were grown at 37°C in a 5% CO_2_ environment in DMEM Glutamax medium (Gibco) supplemented with 1% Pen-Strep and 10% fetal calf serum (FCS).

### Tissue culture models

Respiratory and intestinal tissues are reconstituted from primary human cells collected during surgical procedures. The SmallAir model (24 well plate format) is developed by Epithelix (Geneva, Switzerland) from distal lung epithelial cells as previously described [36], the EpiAirway (96 well plate format), EpiAlveolar (12 well plate format) and EpiIntestinal (96 well plate format) models are developed by Mattek (Ashland, USA) from, respectively, tracheal/bronchial epithelial cells; alveolar epithelial cells, pulmonary endothelial cells and fibroblasts, and finally from small intestine epithelial cells. These models are cultured at the air-liquid interface and fully recapitulate the polarized pseudostratified architecture of human tissues as well as their cellular composition and defense mechanisms as indicated on the companies’ websites (https://www.epithelix.com/products/ and https://www.mattek.com/products/). 3D neural tissues are closed organoid structures engineered by the Neurix company (Neurix Geneva, Switzerland) from human pluripotent stem and contain a mixed population of mature neurons and glial cells (https://www.neurix.ch/copy-of-blastomabrain-1). All tissues were cultured according to manufacturer’s instruction in the provided culture medium.

### Viral stocks

RNA transcription and transfection for viral stock production in HeLa Ohio was performed as described previously [21]. Briefly, after transfection, cells were incubated at 33°C. Seven days after transfection, supernatants and cells were collected, subjected to 3 freeze-thaw cycles, purified by centrifugation to remove cell debris and passaged in 80% confluent HeLa cells. When cytopathic effect (CPE) was observed, supernatants and cells were collected and the process was repeated for a 2^nd^ passage. After the 2^nd^ passage, when CPE was observed, supernatants were collected, subjected to 3 freeze-thaw cycles, purified and aliquoted. Viral stocks were quantified by RT-qPCR with a reference standard as described previously [37] and titrated in Hela Ohio cells. EV-D94 stock contains 4.56E9 RNA copies/ml and 1.12E7 TCID50/ml while EV-D68 stock contains 1.08E9 RNA copies and 3.55E7 TCID50/ml. EV-A71 (Genbank accession number: JN544419; subgenogroup C2) was used as negative control for sialidase assay.

### Human 3D tissues infection

SmallAir (24 well plate format), EpiAirway (96 well plate format), EpiAlveolar (12 well plate format) and EpiIntestinal (96 well plate format) tissues were infected apically with 100μl (24 well plate format) or 20μl (96 well plate format) of medium containing 10^5^ RNA copies/μl of each virus. The viral inoculum was washed out 3 times at 3hpi. After these 3 washes and then every day, 200μl and 100ul, respectively, of medium was applied apically for 20 minutes at 33°C or 37°C for infection run at 37°C, for sample collection. For binding assay, viruses were incubated for 1h at 4°C and tissues were washed 3 times to remove unbound virus before lysis and RNA extraction. For single-step growth curve assay, tissues were lysed directly after inoculation and washes to measure residual virus (0h) and then at the indicated timepoints (Fig 3C–3E) for RNA extraction. 3D neural tissues (MiniBrain) were infected with 10^7^ RNA copies diluted in 20μl of culture medium. Due to tissue fragility, the viral inoculum was not washed out. Tissues were lysed 2hpi and 3dpi to measure cell-associated virus. RNA was extracted and viral replication was quantified by RT-qPCR.

### RNA extraction and real-time RT-PCR quantification

RNA was extracted using the NucliSens easyMAG magnetic beads system (BioMérieux, France) or E.Z.N.A. Total RNA Kit (Omega Bio-tek) according to the manufacturers’ instructions. RT-qPCR was performed using the quantitative Entero/Ge/08 assay (forward primer 5’-GCTGCGYTGGCGGCC-3’, reverse primer 5’-GAAACACGGACACCCAAAGTAGT-3’ and probe 5’-CTCCGGCCCCTGAATGYGGCTAA-3’) as previously described [37] in a one-step format using the QuantiTect Probe RT-PCR Kit (Qiagen, Switzerland) according to the manufacturer’s instructions, in a StepOne Applied Biosystems thermocycler. 10-fold dilution series (from 2.5x10^8^ to 2.5x10^5^ copies/ml) of the *in vitro* transcribed full-length pBMH-EV-D68 were used as a quantitative reference standard for each run. Daily viral production was quantified in apical washes, while cell-associated virus was quantified from whole tissue lysates. mRNA of IFN-λ, INF-β (primers and probes described in [38]) and ISG15 (HS01921425_s1, ThermoFischer) were amplified by TaqMan assay while mRNA for MDA-5 (5’-TGCAGTGTGCTA GCCTGTTC-3’ & 5’-TAAGCCTTTGTGCACCATCA-3’), MX1 (5’-GTTTCCGAAGTGGACATCGCA-3’ & 5’-GAAGGGCAACTCCTGACAGT-3’), IFIT2 (5’-GGGAAACTATGCCTGGGTC-3’ & 5’-CCTTCGCTCTTTCATTTTGGTTTC-3’) and STAT1 (5’-ATGTCTCAGTGGTACGAACTT-3’ & 5’- TGTGCCAGGTACTGTCTGATT-3’) were quantified by KAPA SYBR FAST One-Step qRT-PCR Master Mix (2X) Kit (Kapa Biosystems) and primers were selected from [39]. Gene expression was calculated with the ΔΔCT method versus non-infected controls and normalized to the endogenous reference gene RNase P (TaqMan RNase P Detection Reagents Kit, Applied Biosystems).

### Western blot

Infected tissues were lysed with RIPA buffer at 2 dpi. 15μg of lysed proteins were loaded on a 10% SDS-PAGE gel and then transferred onto PVDF membrane (Bio-Rad). The membranes were first blocked with 5% skim milk (AppliChem) in TTBS (10 mM Tris HCl, pH 7.5, 500 mM NaCl, 0.05% Tween 20) at RT for 30min and then incubated with primary Ab overnight at 4°C. The membranes were then washed thrice with TTBS and incubated with secondary antibodies conjugated to HRP at RT for 1h. The membranes were washed and later developed with Western Bright Sirius ECL system (Advansta Inc.) for 2 min. Immunoblot images were acquired using Fujifilm LAS 4000 luminescence imager. Primary antibodies used in Western blot include anti-Stat1 (612233, BD), -MDA5 (ENZ-ABS299-0100, Enzo Life Sciences), -MX1 (ab95926, Abcam), -IFIT2 (ab113112, Abcam) and -actin (MAB1501, Merck Millipore). All primary antibodies were diluted 1:1000 except MX1 diluted in 1:2000. Goat anti-rabbit (7074, Cell Signaling Technology) and horse anti-mouse (7076, Cell Signaling Technology) secondary antibodies conjugated to HRP were diluted in 1:5000 and used for chemiluminescence development.

### Immunofluorescence of SmallAir tissues

At 1dpi, tissues were washed 3 times with PBS and fixed with 4% paraformaldehyde at room temperature (RT) for 15 minutes. Tissues were then washed 3 times and permeabilized with Perm/wash buffer (BD, USA), then (after 3 more washings) the first antibody was added and incubated for 1 hour at 37°C. Viral staining was performed using the mAbJ2 diluted 1/500 whereas ciliated cells were stained with the rabbit anti-beta IV tubulin antibody (179504 Abcam, UK, diluted 1/250) and basal cell with anti–p63-α antibody (13109; Cell Signaling, Danvers, Mass). The Alexa Fluor 594-goat anti-rabbit antibody (R37117, Life technologies, USA) and the Alexa Fluor 488-goat anti-mouse antibody (A11029 Life technologies, USA, diluted 1/2000) were used as secondary antibodies and incubated for 1 hour at 37°C. After rinsing with PBS, tissues were stained with DAPI, washed with PBS and mounted onto glass slides in Fluoroprep (BioMérieux, France). Images were acquired with a Zeiss LSM 700 Meta confocal microscope with a 63.6/1.4 objective, processed by Imaris and are presented in 3D projections.

### Sialidase assay

α2–3,6,8,9 Neuraminidase A (sialidase; NEB, USA) was diluted 1:100 in EDB-0.5% BSA buffer (10mM sodium acetate, 0.1M NaCl, 10mM CaCl_2_ and 0.5mM MgCl_2_). Tissues were treated with diluted sialidase for 2h at 37°C. Upon incubation, cells were rinsed thrice with PBS to remove sialidase. Sialic acids were stained using immunofluorescence protocol as previously described, in which biotinylated Sambucus nigra lectin antibody was used as primary antibody (VC-B-1305-M002, Adipogen, diluted 1/250), complemented with Alexa Fluor 568-streptavidin antibody (S11226, Invitrogen, USA, diluted 1/500) as secondary antibody with DAPI counterstain. Images were acquired as described above.

### Transcriptomic analysis

Tissues (2 per condition) were infected as described above, washed extensively and apical samples were collected at 1 and 2dpi. At 2dpi, tissues were lysed and RNA was extracted using RNeasy kit (Qiagen) following manufacturer protocol including the on-column DNase (Qiagen) treatment step. Libraries were prepared with the Illumina TruSeqHT Stranded mRNA protocol. The reads were mapped with the STAR aligner to the human reference genome (hg38) and read-count quantification was extracted with HTSeq. Normalization, differential expression analysis, and enrichment analysis were performed in R programming language with Bioconductor packages. For the analyses, data was normalized in reads per million (rpm) and log2 transformed with a pseudo-count of 1. More precisely, the rpm-normalization was computed by dividing the read counts by the total read count in all genes and multiply by 1,000,000. Fold changes were estimated from the transformed data. We estimate fold changes in expression between two conditions with the formula log2(FC) = log2 (avg_RPM_Intestine_EV68 + 1)—log2 (avg_RPM_Intestine_Mock + 1) where avg_RPM_Intestine_EV68 is the average gene expression (expressed in Read Per Million) in condition Intestine_EV68 and avg_RPM_Intestine_Mock is the average gene expression (expressed in Read Per Million) in condition Intestine_Mock. This formulation mitigates estimated fold change of low expressed genes (e.g. a gene whose expression rise from 0 RPM to 1 RPM obtains a log2(FC) = log2(2)–log2(1) = 1). p-values were not computed on the significance of the computed difference for a given gene, as it would requires assumption on the distribution of the values. We rely on the estimated log2(FC) computed by the above formula and consider genes where log2(FC) exceed 0.5. Gene annotations were obtained from uniprot keywords and reactome.org database [40,41]. Only annotated genes were considered for the enrichment analysis, and p-values were calculated with hypergeometric tests. Raw sequencing files and read-mapping have been deposited in GEO database with accession number GSE184488.

### Neutral red assay

Virus stocks were propagated in cells supplemented with 5 μg/ml neutral red (NR) dye (Alrich, Germany) to generate NR-labelled viruses. SmallAir tissues were infected apically with 10^5^ RNA copies/μl NR-labelled virus as described in previous method. At 0, 1, 2 and 3hpi, one of the infected tissues was exposed to light for 1h while negative controls were kept in dark throughout the experiment. Infected tissues were further incubated up to 48hpi and the apical washes were collected to quantitate viral viruses using RT-qPCR.

### Statistics

Results are expressed as mean (± SEM) and experiments were done at least in biological duplicates. Statistical significance was calculated using ordinary one-way ANOVA (no matching), two-way ANOVA (no matching), with multiple comparisons or unpaired t-tests using GraphPad Prism 7.02 software. *P< 0.05, **P< 0.01, ***P< 0.001, ****P< 0.0001.

## Supporting information

S1 Fig EV-D68 and EV-D94 tropism and IFN-λ induction in 3 different respiratory tissue culture models. The models are derived from human upper (EpiAirway) (A) and lower (SmallAir) (B) respiratory tract and from primary alveolar epithelial cells co-cultured with pulmonary endothelial cells, fibroblasts and THP-1 macrophages (EpiAlveolar) (C). S1B and S1E Fig are also represented in Fig 2A and 2D. In the three models, tissues were inoculated with comparable viral loads for EV-D94 and EV-D68 and replication was assessed by RT-qPCR. Tissues were inoculated apically and washed 3 times at 3hpi. Residual virus was quantified after the 3 washes. Apical samples were then collected at the indicated time points for viral RNA quantification. IFN-λ mRNA levels (D-F) were measured in tissue lysates collected 3dpi and fold change relative to non-infected tissues was calculated with the ΔΔCt method. Significance between viruses is shown with black stars while in D, E and F, significance relative to mock-infected is shown with grey stars.
(TIF)

S2 Fig **Immunostaining of ciliated cells and EV-D94 (A) or EV-D68 (B) in respiratory tissues.** Confocal images of co-staining of viral RNA (J2 antibody–green) and ciliated cells (beta IV tubulin antibody–red). Z-stacked pictures were acquired on the basal side of the tissue (on the left) and the apical side of the tissue (right side).
(TIF)

S3 Fig **Immunostaining of basal cells and EV-D94 (A) or EV-D68 (B) in respiratory tissues.** Confocal images of co-staining of viral RNA (J2 antibody–green) and basal cells (P63 antibody–red). Z-stacked pictures were acquired on the basal side of the tissue (on the left) and the apical side of the tissue (right side).
(TIF)

S4 Fig Immunostaining of tissues treated with sialidase or with control buffer. Respiratory tissues were digested with sialidase for 2h and then processed for immunofluorescence staining. Two representative images of mock- and sialidase-treated tissues are displayed at 60× magnification with sialic acids stained in red and cell nuclei in blue.
(TIF)

S5 Fig Neutral red assay on respiratory tissues. Respiratory tissues were infected with EV-D94 and EV-D68 labelled with neutral red dye and exposed to light at indicated timepoints. Apical supernatants were collected at 48hpi and viral RNAs were quantified by RT-qPCR. Statistical significances are shown relative to no-light control.
(TIF)

S6 Fig Genes differentially expressed in respiratory and intestinal tissues in response to EV-D68 and EV-D94 infection. A) EV-D94-infected respiratory tissues, B) EV-D94-infected intestinal tissues, C) EV-D68-infected respiratory tissues, D) EV-D68-infected intestinal tissues. Yellow and purple dots represent upregulated and downregulated transcripts respectively. Fold change (FC) compared to the respective mock-infected tissue is expressed in log2. Genes are organized in categories based on reactome database. Genes annotated in several reactome categories are repeated in each category. 1* Organelle biogenesis and maintenance, 2* Extracellular matrix organization, 3* DNA Replication, 4* Chromatin organization, 5* Cell-Cell communication, 6* Circadian clock, 7* Digestion and absorption, 8* Muscle Contraction, 9* Reproduction. Functional annotations were obtained from reactome.org. A clearer version of this figure is available at [http://genebrowser.unige.ch/enterovirus/].
(TIF)

S7 Fig Fold change (FC) in log 2 relative to mock-infected tissue of selected genes from Fig 5B. (TIF)

S8 Fig Response of small airway (A) or small intestine (B) tissues infected with EV-D94 and EV-D68 for two days. Tissues were infected with 1E7 RNA copies of EV-D94 (equivalent to 2,46E4 TCID50) and 1E7 RNA copies of EV-D68 (3,3E05 TCID50) and infected tissues were lysed at 2dpi. In panel Aa) and Ba), EV viral loads were quantified by RT-qPCR. In Ab) and Bb) innate immunity transcripts were measured by RT-qPCR from tissues lysed at 2dpi and fold changes were calculated relative to mock-infected controls (ctrl) with the ΔΔCT method. In all panels, statistical significance was calculated relative to EV-D68 infection with 1E7 RNA copies. In Ac) western blot was performed on the day-2 lysates to confirm observations made at the mRNA level.
(TIF)

S9 Fig Response of small airway tissues infected with EV-D94 and decreasing doses of EV-D68. Tissues were infected with 1E7 RNA copies of EV-D94 (equivalent to 2,46E4 TCID50) and 1E7 RNA copies of EV-D68 (3,3E5 TCID50) as well as decreasing doses of the latter. A and B: Viral loads were quantified by RT-PCR from apical wash samples collected at the indicated time points (A) or from tissues lysed at 2dpi (B). C: Innate immunity pathways were measured by RT-qPCR from tissues lysed at 2dpi and fold changes were calculated relative to mock-infected controls (ctrls) with the ΔΔCT method. In all panels, statistical significance was calculated relative to EV-D68 infection with 1E7 RNA copies.
(TIF)
