# Supplementary figures and images for: Comparison of tissue tropism and host response to enteric and respiratory enteroviruses
Source: PLoS Pathog. 2022 Jul 5;18(7):e1010632. doi: 10.1371/journal.ppat.1010632 (PMC9286751; doi:10.1371/journal.ppat.1010632)

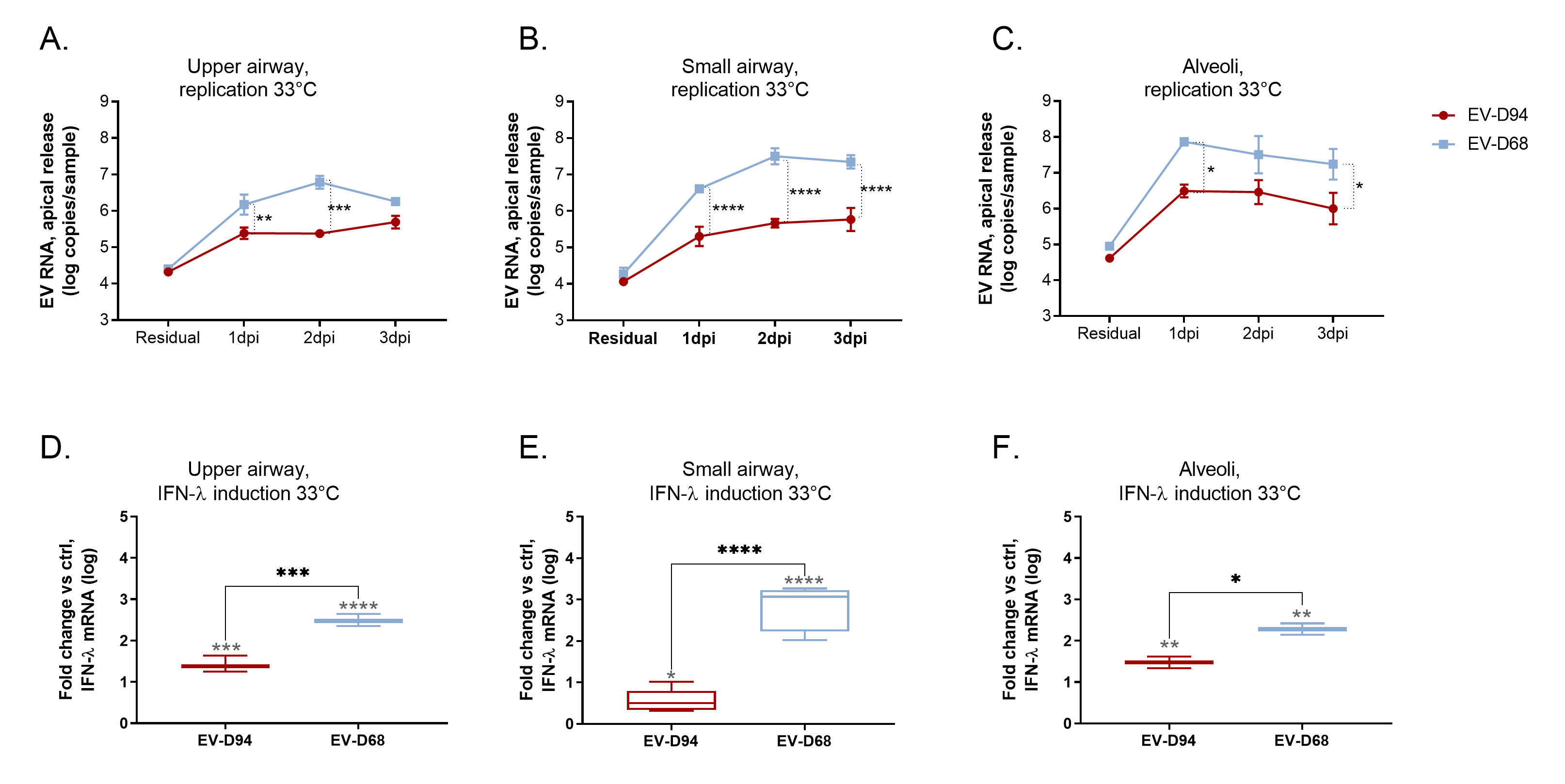

Supplement: S1 Fig — The models are derived from human upper (EpiAirway) (A) and lower (SmallAir) (B) respiratory tract and from primary alveolar epithelial cells co-cultured with pulmonary endothelial cells, fibroblasts and THP-1 macrophages (EpiAlveolar) (C). S1B and S1E Fig are also represented in Fig 2A and 2D. In the three models, tissues were inoculated with comparable viral loads for EV-D94 and EV-D68 and replication was assessed by RT-qPCR. Tissues were inoculated apically and washed 3 times at 3hpi. Residual virus was quantified after the 3 washes. Apical samples were then collected at the indicated time points for viral RNA quantification. IFN-λ mRNA levels (D-F) were measured in tissue lysates collected 3dpi and fold change relative to non-infected tissues was calculated with the ΔΔCt method. Significance between viruses is shown with black stars while in D, E and F, significance relative to mock-infected is shown with grey stars. (TIF) [file ppat.1010632.s001.tif]

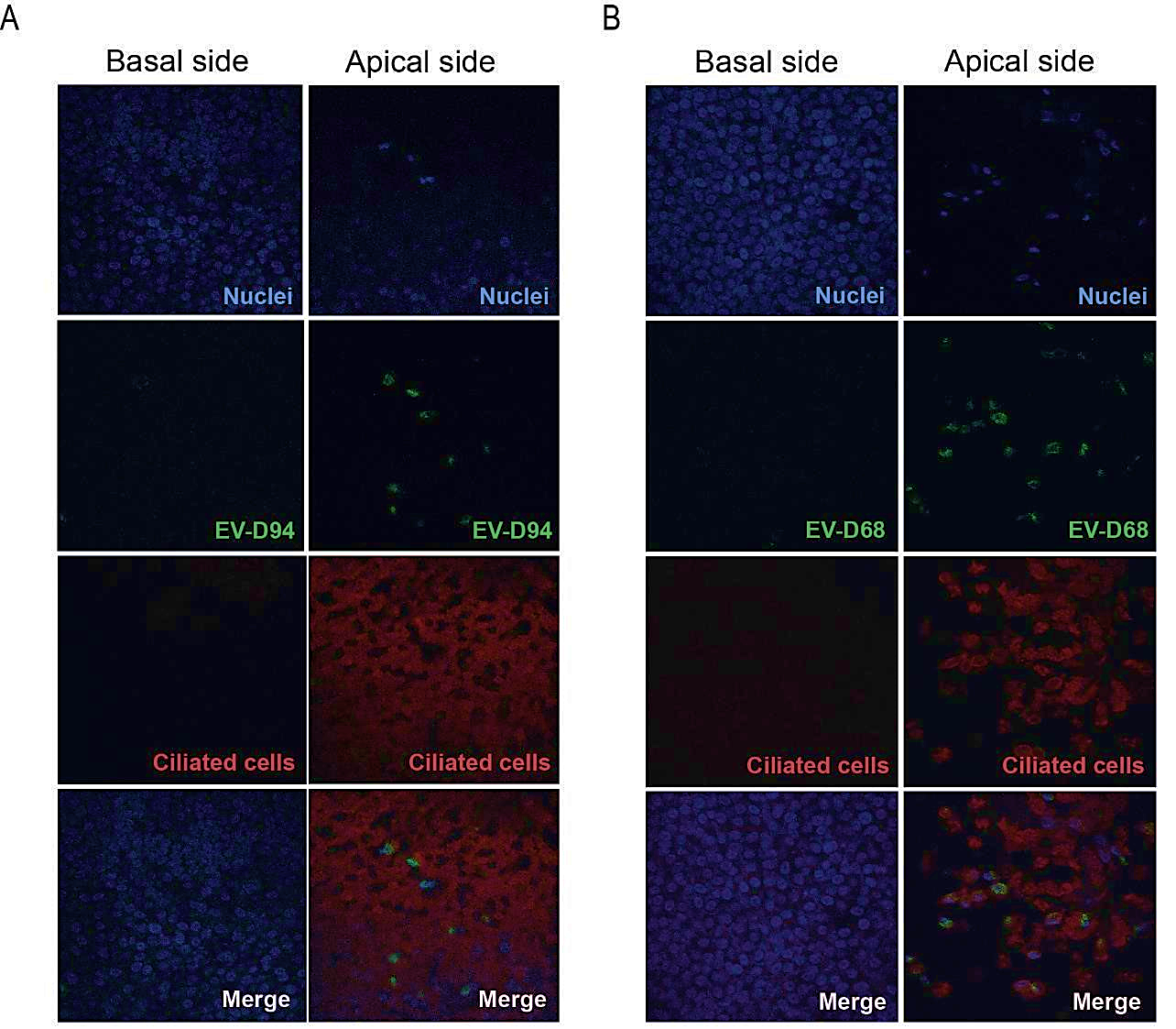

Supplement: S2 Fig — Immunostaining of ciliated cells and EV-D94 (A) or EV-D68 (B) in respiratory tissues. Confocal images of co-staining of viral RNA (J2 antibody–green) and ciliated cells (beta IV tubulin antibody–red). Z-stacked pictures were acquired on the basal side of the tissue (on the left) and the apical side of the tissue (right side). (TIF) [file ppat.1010632.s002.tif]

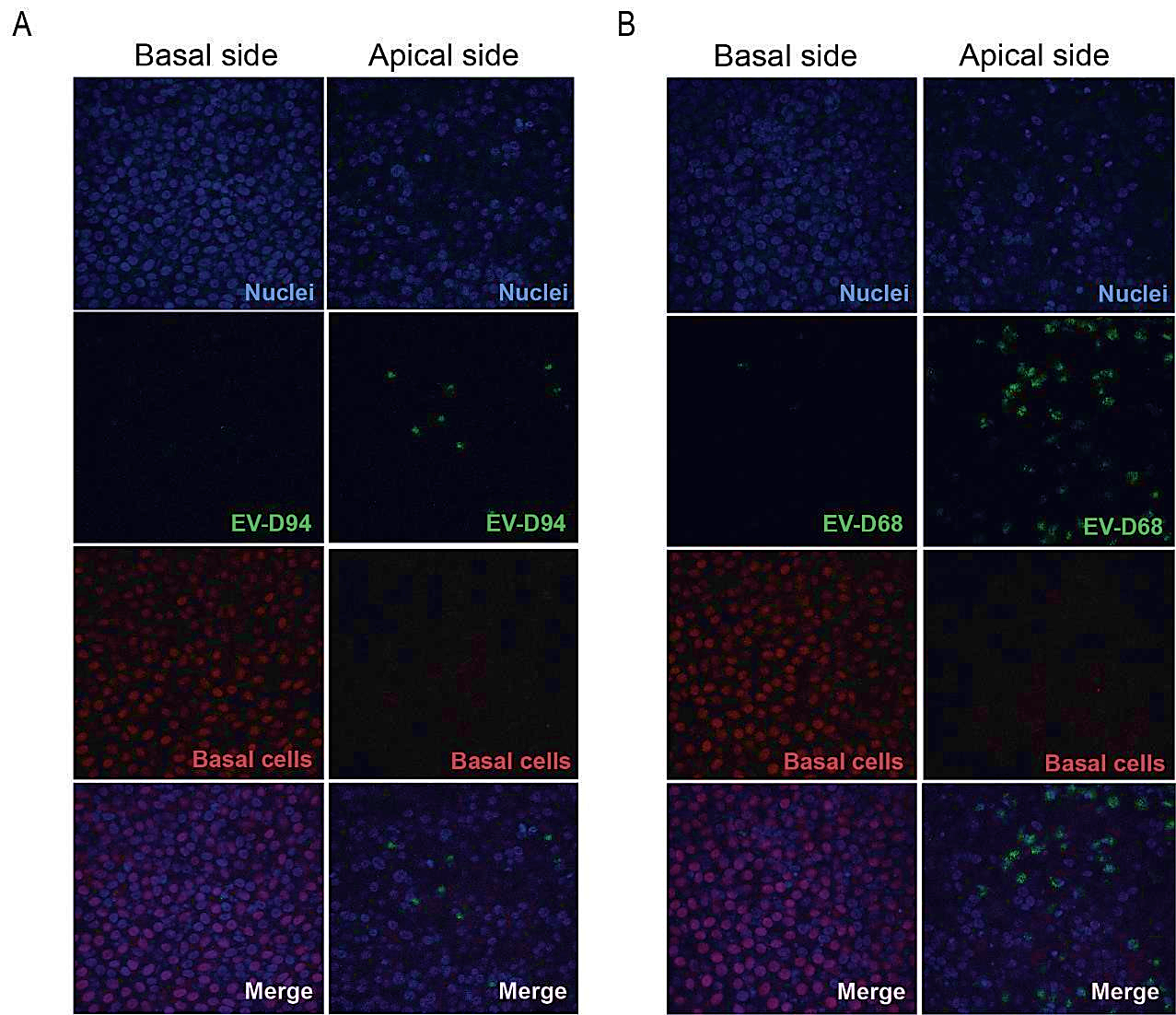

Supplement: S3 Fig — Immunostaining of basal cells and EV-D94 (A) or EV-D68 (B) in respiratory tissues. Confocal images of co-staining of viral RNA (J2 antibody–green) and basal cells (P63 antibody–red). Z-stacked pictures were acquired on the basal side of the tissue (on the left) and the apical side of the tissue (right side). (TIF) [file ppat.1010632.s003.tif]

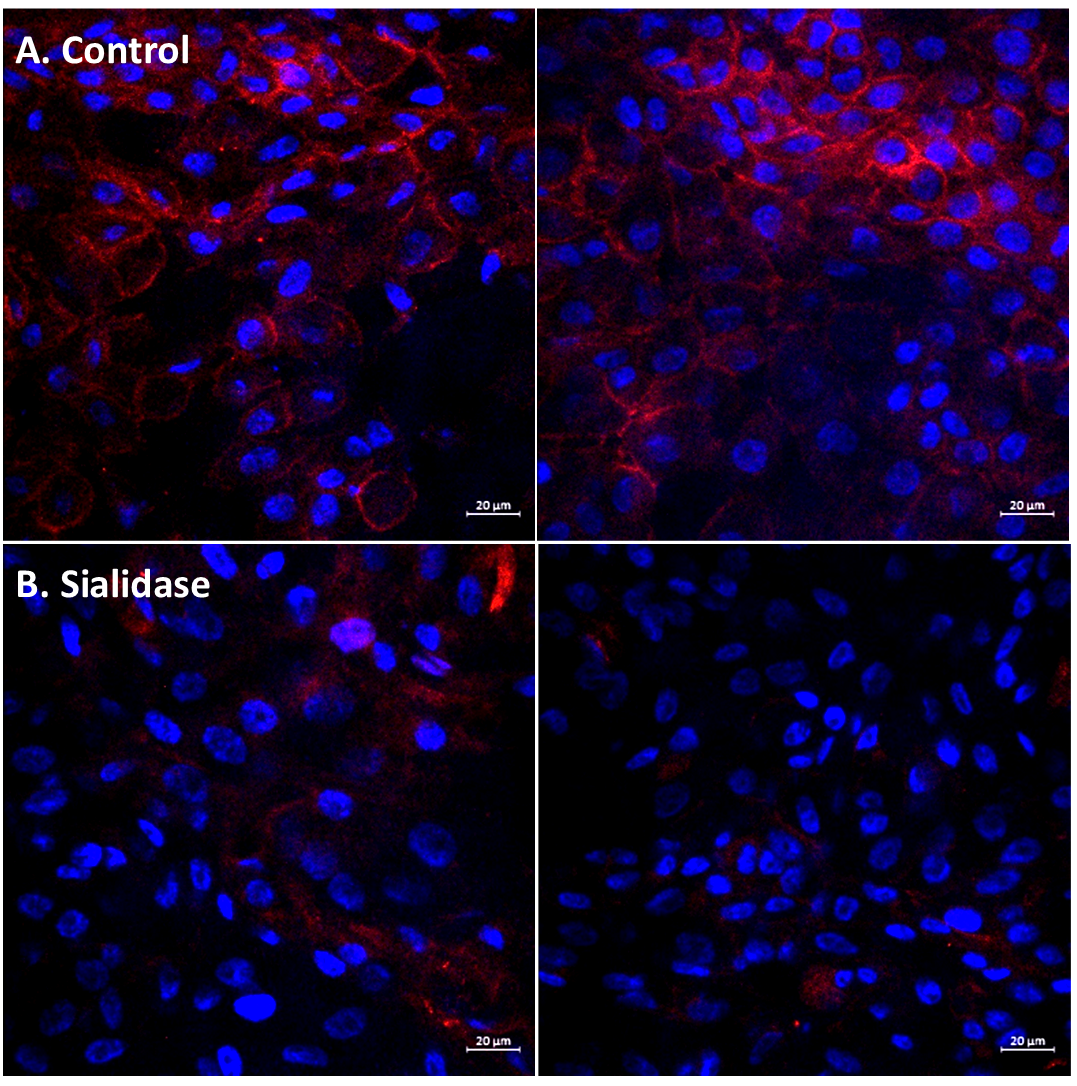

Supplement: S4 Fig — Respiratory tissues were digested with sialidase for 2h and then processed for immunofluorescence staining. Two representative images of mock- and sialidase-treated tissues are displayed at 60× magnification with sialic acids stained in red and cell nuclei in blue. (TIF) [file ppat.1010632.s004.tif]

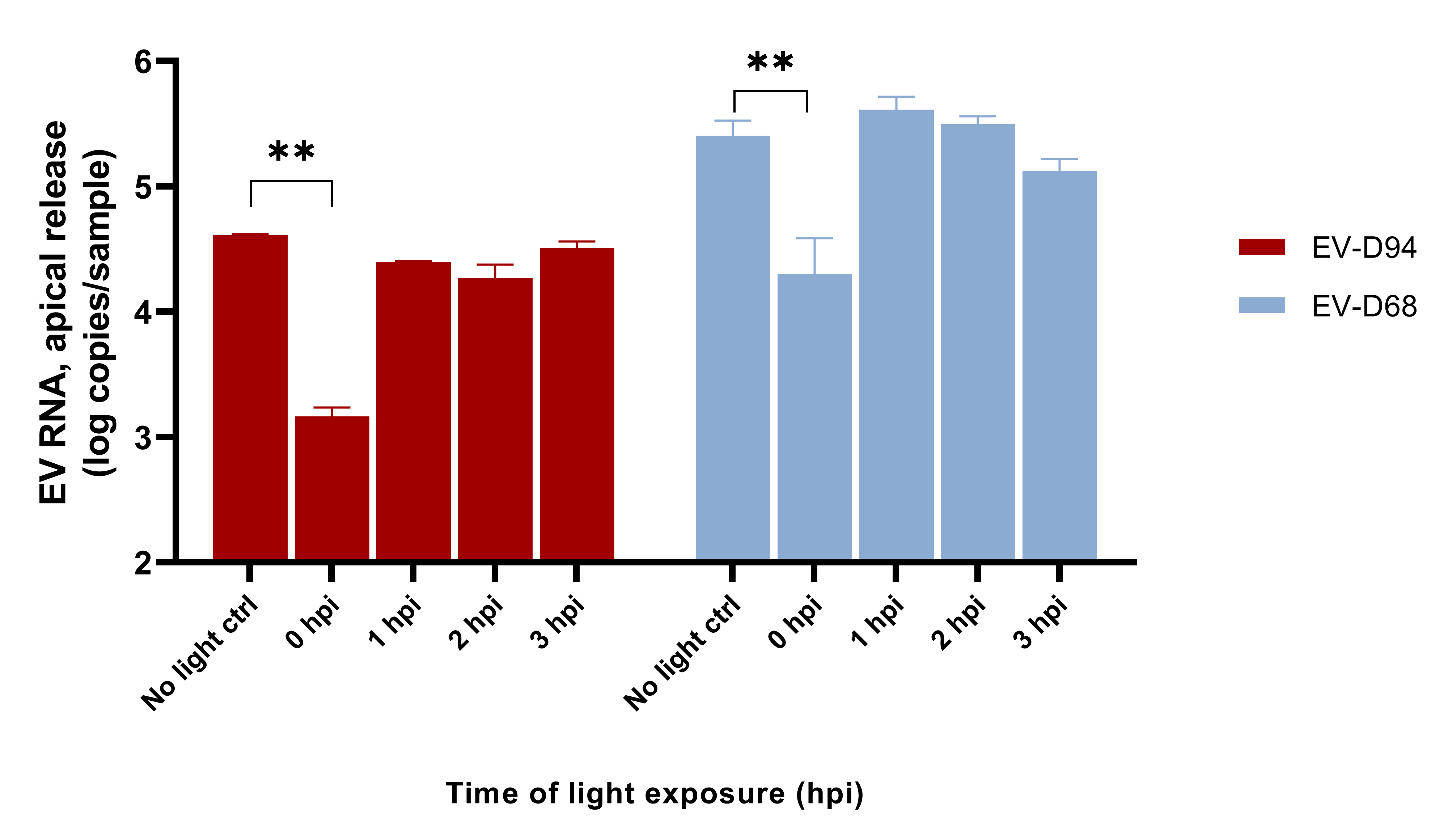

Supplement: S5 Fig — Respiratory tissues were infected with EV-D94 and EV-D68 labelled with neutral red dye and exposed to light at indicated timepoints. Apical supernatants were collected at 48hpi and viral RNAs were quantified by RT-qPCR. Statistical significances are shown relative to no-light control. (TIF) [file ppat.1010632.s005.tif]

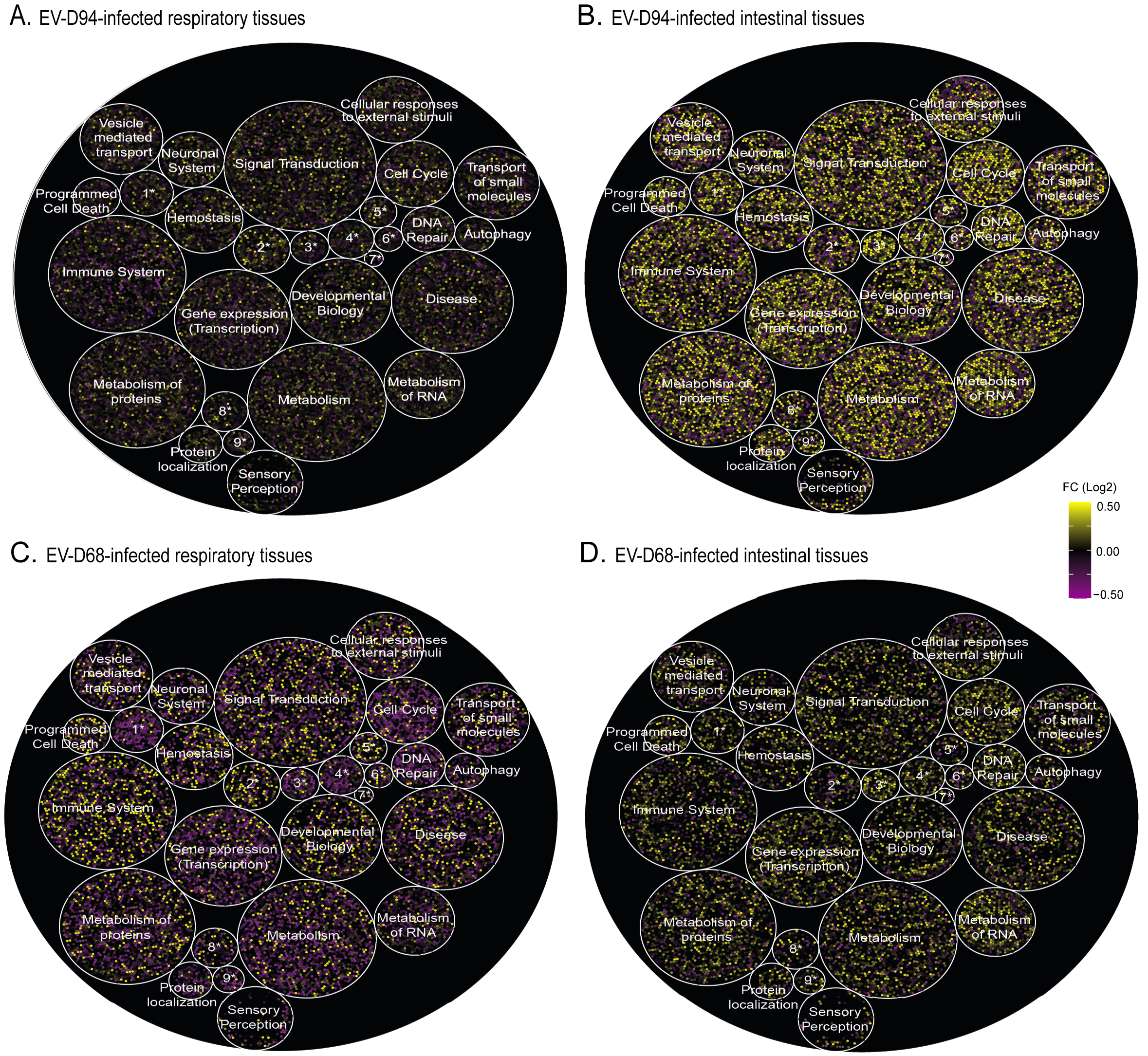

Supplement: S6 Fig — A) EV-D94-infected respiratory tissues, B) EV-D94-infected intestinal tissues, C) EV-D68-infected respiratory tissues, D) EV-D68-infected intestinal tissues. Yellow and purple dots represent upregulated and downregulated transcripts respectively. Fold change (FC) compared to the respective mock-infected tissue is expressed in log2. Genes are organized in categories based on reactome database. Genes annotated in several reactome categories are repeated in each category. 1* Organelle biogenesis and maintenance, 2* Extracellular matrix organization, 3* DNA Replication, 4* Chromatin organization, 5* Cell-Cell communication, 6* Circadian clock, 7* Digestion and absorption, 8* Muscle Contraction, 9* Reproduction. Functional annotations were obtained from reactome.org. A clearer version of this figure is available at [http://genebrowser.unige.ch/enterovirus/]. (TIF) [file ppat.1010632.s006.tif]

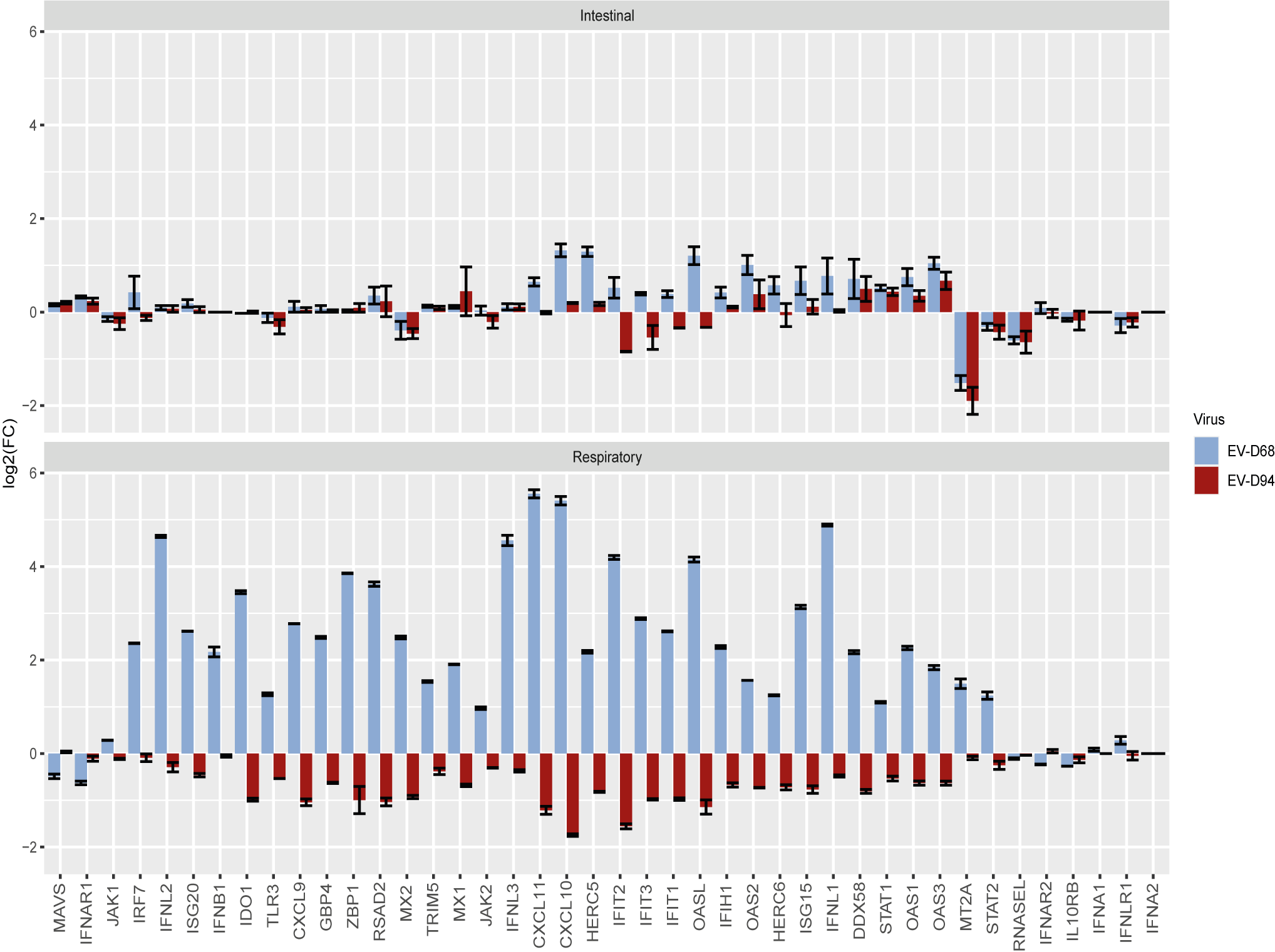

Supplement: S7 Fig — (TIF) [file ppat.1010632.s007.tif]

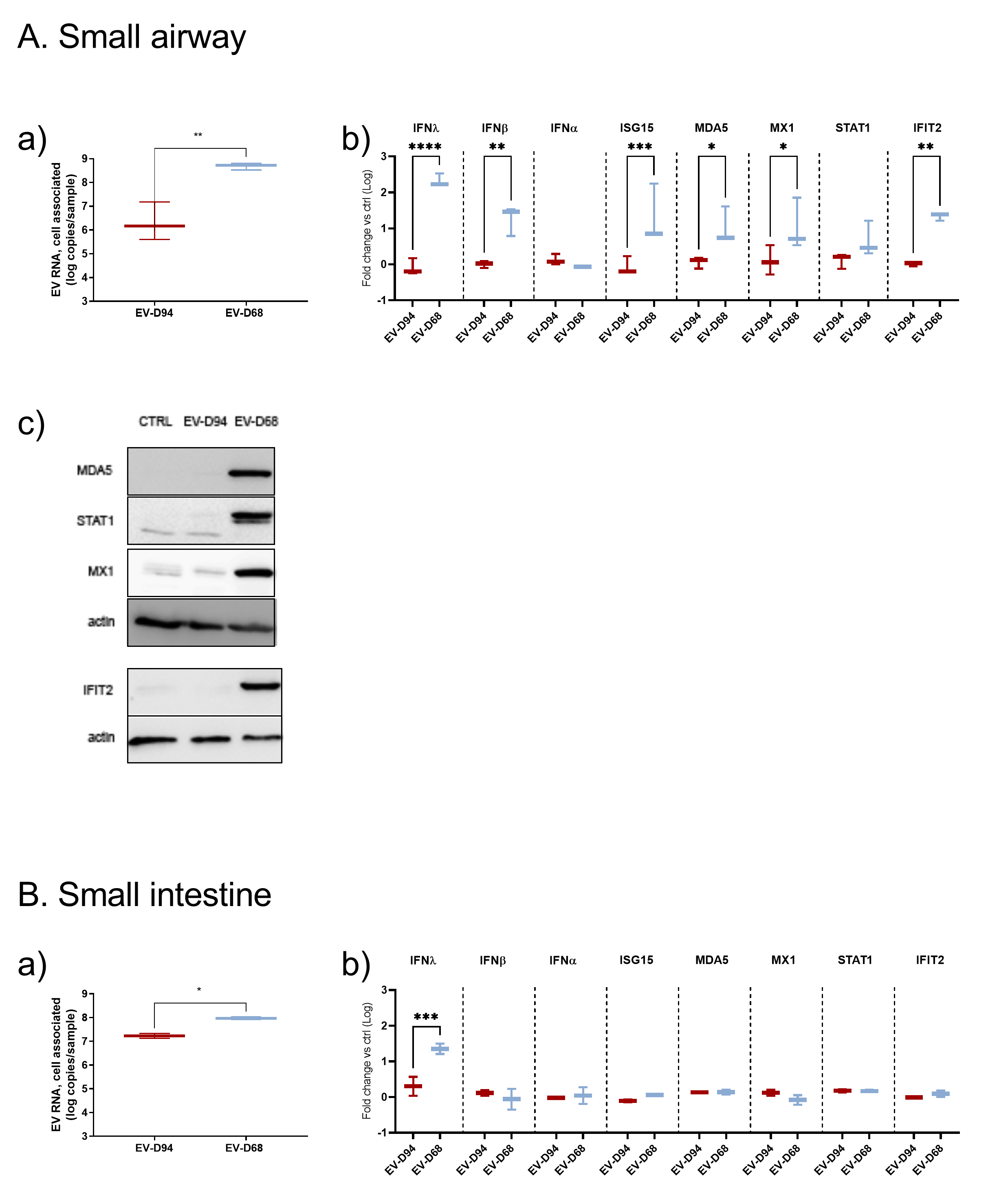

Supplement: S8 Fig — Response of small airway (A) or small intestine (B) tissues infected with EV-D94 and EV-D68 for two days. Tissues were infected with 1E7 RNA copies of EV-D94 (equivalent to 2,46E4 TCID50) and 1E7 RNA copies of EV-D68 (3,3E05 TCID50) and infected tissues were lysed at 2dpi. In panel Aa) and Ba), EV viral loads were quantified by RT-qPCR. In Ab) and Bb) innate immunity transcripts were measured by RT-qPCR from tissues lysed at 2dpi and fold changes were calculated relative to mock-infected controls (ctrl) with the ΔΔCT method. In all panels, statistical significance was calculated relative to EV-D68 infection with 1E7 RNA copies. In Ac) western blot was performed on the day-2 lysates to confirm observations made at the mRNA level. (TIF) [file ppat.1010632.s008.tif]

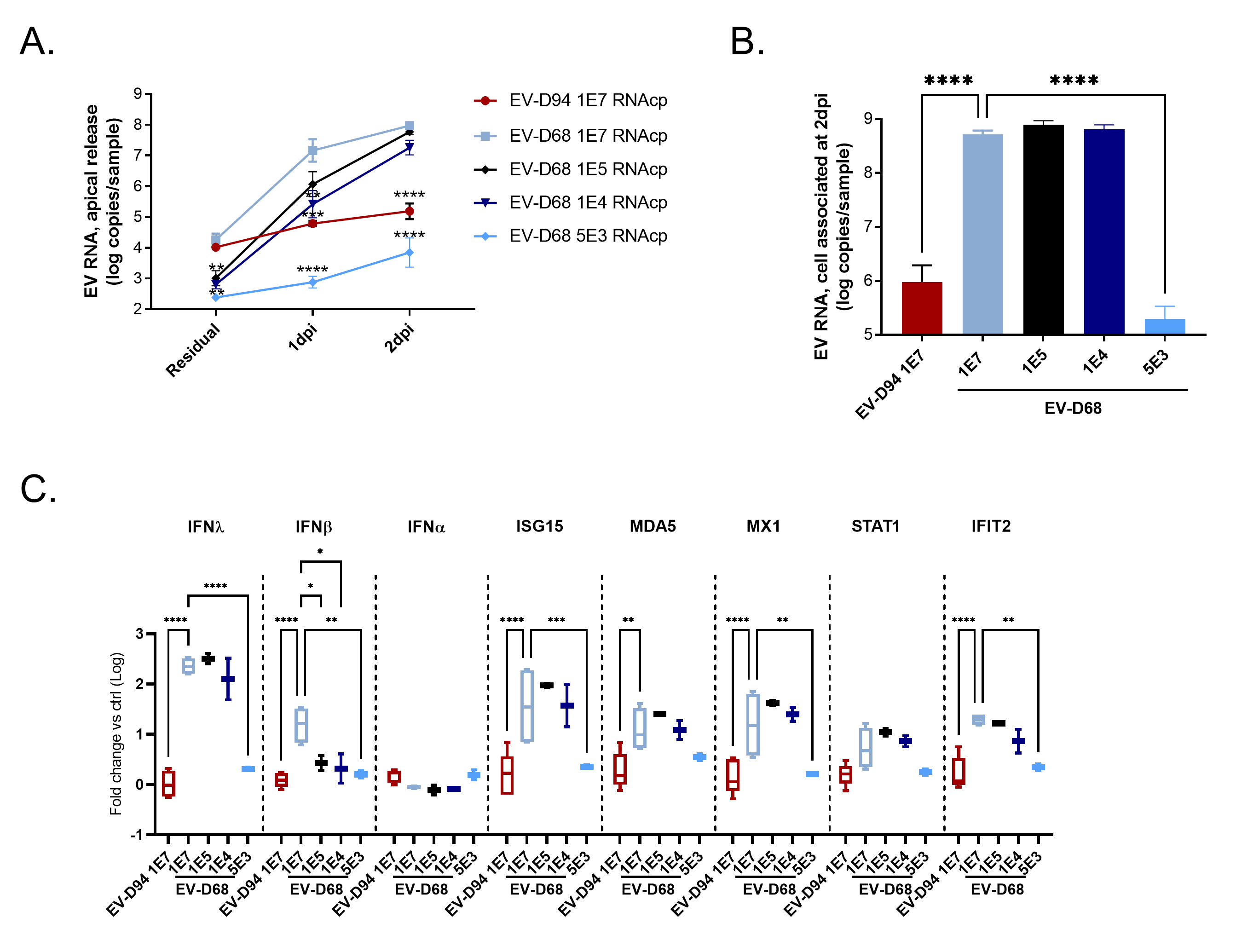

Supplement: S9 Fig — Tissues were infected with 1E7 RNA copies of EV-D94 (equivalent to 2,46E4 TCID50) and 1E7 RNA copies of EV-D68 (3,3E5 TCID50) as well as decreasing doses of the latter. A and B: Viral loads were quantified by RT-PCR from apical wash samples collected at the indicated time points (A) or from tissues lysed at 2dpi (B). C: Innate immunity pathways were measured by RT-qPCR from tissues lysed at 2dpi and fold changes were calculated relative to mock-infected controls (ctrls) with the ΔΔCT method. In all panels, statistical significance was calculated relative to EV-D68 infection with 1E7 RNA copies. (TIF) [file ppat.1010632.s009.tif]
